# Naturalizing pollution: a critical social science view on the link between potash mining and salinization in the Llobregat river basin, northeast Spain

**DOI:** 10.1098/rstb.2018.0006

**Published:** 2018-12-03

**Authors:** Santiago Gorostiza, David Saurí

**Affiliations:** 1 Institut de Ciències i Tecnologia Ambientals, Universitat Autònoma de Barcelona, Barcelona, Spain; 2 Departament de Geografia, Universitat Autònoma de Barcelona, Bellaterra, Catalunya, Spain

**Keywords:** salinization, Llobregat, environmental history, environmental justice, water quality, disinfection by-products

## Abstract

The scientific literature distinguishes between primary or natural and secondary or human-induced salinization. Assessing this distinction is of vital importance to assign liabilities and responsibilities in pollution cases and for designing the best policy and management actions. In this context, actors interested in downplaying the role of certain drivers of human-induced salinization can attempt to neglect its importance by referring to natural salinization, in a similar fashion to other pollution and health-related cases, from tobacco smoke to climate change. Potash mining, which has experienced continued growth during the last decades and is a significant contributor to salinization, is prone to originate such controversies because natural salinization from the saline geological catch can be mixed with salinization produced by mining waste such as brines and mine tailings, thus obscuring the distinction between causes. By reviewing the long-standing social and environmental conflict caused by potash mining in a region of Mediterranean climate—the Llobregat river basin—in this article, we highlight the importance of the impacts of salinization on human health and provide a critical social science perspective on salinization processes.

This article is part of the theme issue ‘Salt in freshwaters: causes, ecological consequences and future prospects’.

## 1. Introduction

How can we best understand the causes of primary and secondary salinization? Why and how is it possible to naturalize a human-induced process? What would be an alternative framework for fully discerning the social and political nature of salinization and especially of secondary salinization? In attempting to unravel these questions, the social sciences, and most notably, studies seeking to develop interdisciplinary approaches to environmental problems, may be of interest. This has been recognized in major scientific journals such as *Science* [1] and *Nature* [2] although the specific character of the collaboration between natural sciences, social sciences and humanities remains open to discussion [3]. At any rate, the reduction of all the complex relationships between nature and society to a black box labelled ‘human activities’ typical of early Earth System models [4] is increasingly called into question. For some authors, leaving outside the multiple human and social dimensions and their relationships would be equal to ‘designing a bridge without accounting for traffic’ [2, p. 365].

Bringing the social sciences in may help in improving the understanding of human agency and therefore contribute to designing better policy actions to manage environmental change. Social science focuses, among others, on issues related to identity, difference, justice, ethics and equality [5]. In relation to environmental matters, social science would be interested in the question of ‘who gets what kind of resources (or hazards), where, and how’ and what are the implications of this for both nature and society alike. However and contrary to the more unified scientific corpus of the natural sciences, social sciences are characterized by more pluralistic views [6].

In the field of water, two social science communities can be identified [7]. First, sociohydrology attempts to become a new science for humans and water [8]. Its aim is the integration of the structure and dynamics of natural and social systems; the outcomes in terms of well-being for humans and non-humans, and the values and norms necessary to reach those outcomes [8,9]. For sociohydrologists, salinization would be an example of a process deeply altered by human behaviour and the ideal research design would be one attempting to identify, probably by developing a model, the whole range of these human behaviours leading to increasing concentrations of salt in freshwaters. This model then could be integrated with other models that explore the physical and biological sides of salinization in order to produce new knowledge for sustainability goals. Second, the hydrosocial perspective [7] would define salinization as a phenomenon constituted by multiple factors originating in both the human and the non-human world but the consideration of ethics, equity and justice would play a central role in the research under the fundamental idea that salinization and its effects would not be experienced equally by those affected. At a deeper level, critical environmental social science research, that is, research centred upon issues of inequality and power in the distribution of environmental costs and benefits, also questions the separation between nature and society because this separation is seen as an obstacle to fully comprehend many environmental problems and their possible solutions [10].

Regarding freshwater salinization, recent research has called for a better integration of the insights of natural and social sciences towards a more complex understanding of salinization processes [11]. River salinization is caused by a diverse array of drivers, both natural and anthropogenic. Research within the natural sciences has traditionally distinguished between primary or natural salinization and secondary or anthropogenic salinization. Natural salinity of rivers is related to the geology of catchments, the proximity of the sea, vegetation, topography and climate throughout time. Secondary or anthropogenic salinization can be caused by a wide bundle of causes including agriculture, mining, industrial activities or use of salt for water de-icing, among others [12].

Therefore, distinguishing between natural and anthropogenic salinization—as well as within the different drivers of human-induced salinization—is of vital importance for the different actors affected by these processes also in different ways, because it represents a necessary step in assigning liabilities and responsibilities in pollution cases, under the ‘Polluter Pays’ principle (EU Directive 2004/35/EC). In this context, actors interested in downplaying the role of certain drivers of human-induced salinization can attempt to neglect its importance and divert attention by referring to natural salinization. Downplaying causal factors supported by scientific evidence is not limited to salinization but involves other cases of contamination. By successfully ‘naturalizing’ pollution—that is, presenting a human-induced process as natural, beyond human control—actors can attempt to erase socioenvironmental struggles and achieve immunity from social and political scrutiny, thus avoiding legal responsibilities emanating from the ‘Polluter Pays’ principle and therefore shifting away environmental and social costs [13–15]. Similar examples abound in many health and pollution cases, from the contamination caused by nuclear weapons production to climate change, where attempts have been made to exonerate the supposedly guilty parties by downplaying human causation [14,16,17]. This article examines the case of the naturalization of human-induced salinization in historical perspective.

The paper is organized as follows. The next section discusses the social impacts of human-induced salinization, with particular attention to the potential effects of salinization on human health. Following this, we examine the case of the salinization of the Llobregat River (northeast Spain) since the 1920s to the present day, focusing on the discussions about natural and human-induced salinization. Finally, in the conclusions, we point at the importance of an interdisciplinary approach in controversies where causal factors supported by scientific evidence are downplayed, such as the naturalization of pollution.

## 2. Potash mining and social impacts of salinization

Several human activities such as irrigation for agriculture, industrial activities, use of de-icing salts or mining contribute to the growing global problem of freshwater salinization. Among the different mining activities contributing to salinization, potash extraction stands as critical. ‘Potash’ refers to a wide variety of mined and manufactured salts containing potassium in water-soluble form, but also chloride, sulfate and other compounds. Its production predominantly focuses on the mining of evaporitic salt deposits, and as a result, potash mine wastes are always saline. Potash is mostly used as a fertilizer, but it also has industrial and chemical applications. Its use and importance continue to grow. World production increased by 20% between 2012 and 2016, reaching 39.67 million metric tons, while demand for all uses is projected to reach 45.6 million tons by 2021 [18–20].

Potash ores rank among the major producers of waste of all salt ores. The extraction of potassium generates both liquid and solid waste—brines and tailings—which can be backfilled into the underground mines but are often disposed in different ways. Solid tailings are usually piled near the mine site, creating large mounds of sodium chloride. If not properly impermeabilized, these tailings contribute to salinization when rainfall dissolves salts and runoff flows into streams. Brines are piped to be discharged into the ocean or collected and treated in ponds, to be later released into local rivers [20].

### (a) The Werra and the Llobregat: two rivers of salt

The impact of potash mining in freshwater salinization has been well documented in Europe [21]. The increase of salinization caused by potash mines and its effect in water quality and ecosystems has been particularly well studied in two regions where this activity has greatly affected the nearby rivers for the past decades. Both the Werra River in Germany and the Llobregat River in northeast Spain constitute interesting case studies for analysing how salinization has unfolded as a historical process, inevitably confusing primary and secondary salinization sources in both rivers. In the case of the Werra, potash mining and processing activities in Thuringia and Hesse highly increased the salinity of the river between 1950 and 1970, reaching in its lower part salinities higher than those of the North Sea, and modifying the flora and fauna of the river [22–24]. After the implementation of a programme to control salinity in the year 2000, salinization has significantly decreased, allowing for a partial recovery of the flora [25]. Quite similarly, in the Llobregat catchment, potash mining started in the 1920s and soon contributed to a major increase in the salinization of the river [26].

The impacts of salinization in the Llobregat ecosystems have been studied since the 1990s and have recently received renewed attention [27–29]. Since the 1990s the quality of waters has significantly improved upstream of the potash mines but shows little progress in its biological conditions downstream of the mining areas [27]. The most recent studies have found conductivities nearly three times higher than seawater, caused by salt leachates from the mine tailings, therefore underlining the serious ecological damage caused by potash mining to the surrounding environments [28]. These studies, however, have mostly focused on the impacts of salinization in ‘nature’, paying little attention to its social costs to human health and public infrastructures, despite the impacts that potash mining caused in rivers such as the Werra or the Llobregat, vital for the water supply of Bremen [22] and Barcelona [26], respectively. Another significant social dimension of the conflict caused by salinization in the two cases concerns the thousands of jobs provided by mining activities.

The case of the Llobregat stands out as particularly relevant in relation to the mix of natural and anthropogenic causes of salinization. Owing to the saline geology of the Llobregat river basin, primary salinization and secondary salinization historically have been mixed. As a result of the saline geology of the catchment, salt concentrations in its waters were significant even before the potash mines started their activity [30]. In fact, as observed by locals and visiting naturalists, salt concentrations could reach critical levels during the flash floods of the Cardener River, one tributary of the Llobregat located in areas with higher saline geology [30,31]. For centuries, salt has been exploited in the region, becoming its main resource. However, after potash salts were discovered in 1912 and production started in the 1920s, salinization caused by this anthropogenic activity was added to existing natural salinity factors. Claims about the social impacts of secondary salinization and discussions about its relative importance in relation to the ‘natural’ salinization started soon thereafter and remain controversial to this day, when they are discussed in European courts of justice [26]. As a result of these controversies, a significant amount of historical data has been collected for the Llobregat River and its tributaries.

The Llobregat River is also relevant because it constitutes an example of the Mediterranean climate [32]. In Mediterranean streams with prolonged droughts and highly variable flows, the impacts of potash mining in freshwater salinization can be particularly difficult to control. During the dry season or during prolonged droughts, the limited dilution capacity of these rivers may lead to increased salinity. When salts pile up as mine tailings are diluted and washed into river waters after heavy precipitation and flooding, sudden increases of salinity may occur as well [21]. In a context of global warming, with lower river discharges predicted for southern Europe, the already limited dilution capacity of Mediterranean streams is likely to decrease, therefore expanding the regions affected by salinization [12].

### (b) River pollution and the potential impacts of salinization on human health

The history of pollution may offer some interesting examples as to how different human and non-human components intervene in creating the circumstances for harmful episodes to occur affecting the human and the non-human world. The drift between nature and society, especially from the seventeenth century onwards coincides with increasing manifestations of what has been dubbed as the ‘anger of nature’ [33, p. 199] under various forms of harm from pollution and toxicity inflicted on the human individual supposedly liberated from the constraints of nature. Nature may have been transformed by human action but in this process, it retained the agency to continue to influence human affairs through new (and dangerous) environmental hazards [34].

An example of how different configurations of human and non-human agents participate in the debates on pollution is provided by Garcier [35] in his account of river pollution in eastern France in the nineteenth and early twentieth centuries. In the nineteenth century, organic matter was considered the worst form of pollution for its immediate effects on human health through water-borne diseases. Garcier explains how in Lorraine, dangerous bacteria originating in organic waste were eliminated by the antiseptic effect of chlorides and heavy metals discharged in rivers by mining and industrial activities. Mining and industrial interests succeeded therefore in legitimizing toxic discharges into rivers because of their ‘beneficial’ effects in pollution control. The clash between networks of agents present in the two forms of pollution was solved in favour of mining and heavy industry probably because organic waste was constructed as dangerous for human health, while salts and heavy metals were constructed as the opposite [35].

In other parts of the globe, however, toxic chemicals and not organic matter were the immediate source of danger and harm. Nonetheless, denial of involvement by mining and industrial activities in pollution episodes was widespread. The case of the Ashio mine in Japan in 1906 and the ‘it hurts, it hurts’ disease caused by toxic flows represents an example of how flooding (a natural process) and not copper mining was considered the main cause of the ‘bad water’ ruining agricultural fields and killing peasants and their families [34]. In this sense, history and especially environmental history—usually defined as ‘the history of the mutual relations between humankind and the rest of nature’ [36, p. 6]—can shield from the perils of naturalization by critically examining how the process of naturalizing pollution has unfolded through time. In the case of salinization processes, the analysis of assemblages of agents and of their historical changes may reveal hidden relations pointing towards human responsibilities and may be also of interest when approaching the subjects of causation and uncertainty, both key factors in naturalizing pollution [37].

Literature within the natural sciences has generally focused on the impacts of salinization in flora and fauna, mostly leaving aside the social impacts of salinization. So far, research on the impacts of salinization on human health has pointed to the danger of hypertension among populations exposed to an increase of water salinity related to saltwater intrusions caused by rising sea-levels [38–42]. Researchers underline the diverse and long-term effects that high salinity concentrations in water can cause on human health and warn about the growing impacts of climate change for coastal populations [38–42]. Critical social science research explicitly connects the rise of salinization to environmental justice claims, pointing at the great mismatch between the contribution of climate change-related emissions from certain countries such as Bangladesh, and the burden of the negative consequences suffered by coastal areas in this country [38].

Other impacts of salinization over water quality—and eventually on water supply and human health—may be more complex involving the interaction between specific ions and other pollutants. The wide range of possible interactions has motivated calls for further research in this field [12]. One example of these complex interactions and the potential impact on human health is the contribution of chloride and bromide to the formation of disinfection by-products (DBPs). Starting in 1974 with the identification of trihalomethanes (THMs) [43], which include chlorinated and brominated compounds, several researchers identified hundreds of different substances that are created during the process of water disinfection with chlorine or chloramine [44]. In doing so, they unveiled a paradox: that the very product which water disinfection has relied on for decades—chlorine—also contributes to the creation of other substances potentially harmful to human health [44]. As we present in the following section, this was a key discovery with significant consequences for the control of human water supply both in the Werra and the Llobregat.

## 3. The salinization of Llobregat river from the 1920s to the present day

Early in 2018, the European Commission threatened to bring Spain to the Court of Justice of the European Union (EU) for violating the Extractive Waste Directive (Directive 2006/21/EC) and the Water Framework Directive (Directive 2000/60/EC) [45]. The reason was the serious environmental problems caused by the potash mine tailings in Súria and Sallent, both located in the Llobregat river basin [45]. In the potash mines of the region, more than 3 kg of salt wastes are produced for each kilogram of potassium obtained [46]. As a result, the current major mine tailing—El Cogulló, active since 1977—has become the largest accumulation of waste of Catalonia (northeast Spain), with more than 50 million tons of salts [46]. The EU investigation on potash mining has been ongoing since 2014, starting with a report from local activists, and accusing Spain of endangering the environment and human health in the region by allowing the continuation of the mining activities without proper control measures [47]. While the Spanish government has acknowledged the importance of the pollution caused by potash mining, it also stated that it was difficult to distinguish and calculate how much salinity could be attributed to natural causes and how much to potash extraction [47].

References to ‘uncertainty’ have been recurrent since the beginning of the extractive activity in the 1920s and the emergence of the first protests against the mines. Even if the property of the different mines has changed several times during the last decades, the insistent reference to the technical difficulties to distinguish natural or primary salinization from that caused by the extractive activity has remained, thus contributing to the ‘naturalization’ of the salinization caused by potash mining (see §3a below). Naturalizing pollution involves a discourse that undermines causation and presents pollution as something beyond human control [13–15]. In a similar fashion, the reference to uncertainty and even the denial of human-induced salinity from the mines has been combined during more recent times with a mention of the wide array of sources that contribute to an increase of human-induced salinization in the basin, such as agriculture, increased population growth in the region, etc. While rigorous assessments of the different sources of salinization are needed (see Cañedo-Argüelles and co-workers [48–52]), the socioenvironmental history of the Llobregat River, presented below, illustrates that the salinization caused by potash mining has without doubt influenced the quality of water supply in the region.

### (a) Natural and human-induced salinization

In 1926, soon after the beginning of commercial potash mining in central Catalonia, water users downstream of the Cardener and Llobregat rivers filed complaints against mining companies [26]. Industrial owners claimed that mining companies were taking river water illegally and dumping saline wastewater that damaged the industries' pipes [46]. The topic of salinization increasingly attracted the attention of farmers, fishermen and the water company supplying Barcelona. During the brief democratic period of the Spanish Second Republic [1931–1939], a public commission was established to analyse the impacts of salinization in agriculture, industry and other sectors [32,53]. It also collected information about chlorine concentrations in the region before potash salts extraction started, thus establishing reference levels of ‘natural’ salinization [30,53]. The main recommendation of the commission was to build a brine sewer to the sea thus avoiding the release of mining wastewater to the river [26,53]. In addition, the regional government enacted a Salinity Law that established a legal limit of 250 mg Cl^−^ l^−1^ for river waters and a monitoring system with water control stations downstream of each mine and near the city of Barcelona [54].

Unwilling to acknowledge full responsibility for the increase in salinization, mining companies called these results into question [46]. On the one hand, they argued, salts had always been present in the Cardener and Llobregat waters, as a result of the geologically saline nature of central Catalonia. Natural saline formations could be held responsible for salinization, but in their opinion, there were also other contributing human activities [55]. The increasing population of the cities close to the river Cardener, and their growing water consumption, was targeted as one of these sources of salinity [55]. Moreover, the Barcelona water company was accused of extracting too much water from the aquifers near the sea, causing saline intrusion [26]. Despite the mobilization of a growing social movement denouncing river salinization, conversations about the funding necessary to build a brine sewer and other measures to control the problem stalled [37].

The Spanish Civil War [1936–1939] brought about a completely new scenario which provided critical knowledge against the naturalizing arguments held by the mine companies. As underlined by environmental historian John McNeill, by suspending regular economic activity, armed conflicts may temporarily relieve some of the impacts in the environment—a phenomenon usually illustrated with the recovery of fishing stocks during World War II or by the conservation of certain natural areas located in latent conflict zones [56]. In the case of the Spanish Civil War, the conflict soon caused a decrease in the activity of the potash mines, and extractions eventually came to almost a halt. However, part of the monitoring system put in place years earlier continued functioning, registering a continuous decrease in the salinity of the city waters [57]. Like in an unrepeatable real scale experiment, by the end of the war, the Cl^−^ concentrations in Barcelona's waters were at the lowest levels since the 1920s. The managers of the Barcelona water company celebrated that the naturalization argument had been defeated beyond all doubt [26].

Unsurprisingly, once mining activities resumed under the Francoist dictatorship, river salinization increased again, Republican legislation was cancelled and brine sewer projects shelved [26]. The construction of the first reservoirs in the basin contributed to a better regulation of the river flow and thus to an enhanced control of dilution [37]. Salinization continued growing unabated, but particularly in the 1960s, industrial activity in the Llobregat basin compromised river quality in more aggressive ways [37,58]. By the 1970s, five decades of potash mining in the region had brought salinization to a new ‘normal’, naturalizing the high salinity of the river waters. The Francoist government's approval of a higher legal limit of 350 mg Cl^−^ l^−1^ for river waters validated this situation [26]. In fact, the salinization and poor quality of the Llobregat River waters were key reasons that justified water transfers from other basins to feed the growth of Barcelona metropolitan region [59,60].

The brine sewer project drafted in the early 1930s only became a reality 50 years later and was funded by the regional government of Catalonia [61]. During the 1970s, the presence of chlorine in the Llobregat waters was, on average, above 500 mg Cl^−^ l^−1^, reaching 1300 mg Cl^−^ l^−1^ in 1973 (a particularly dry year) and 1200 mg Cl^−^ l^−1^ in 1979 [61]. The construction of the brine sewer during the 1980s dramatically reduced the presence of chlorine and several ions in the river waters [62]. However, after the extraction activity expanded in the late 1990s, the brine sewer has experienced hundreds of leaks requiring further investments and enlargements [26]. Between 2008 and 2011, the Catalan government invested around 200 € million to alleviate the impacts of potash residues—including 62 € million for the brine sewer improvement and other investments to cover and restore mine tailings [63]. These public investments raised criticisms of concealed public support to the mining company from several fronts and originated new court cases based on a possible breach of the ‘Polluter Pays’ principle (EU Directive 2004/35/EC) [64]. When facing the inquiries of the European Commission, the Spanish State resorted again to the argument about the uncertain distribution of natural and anthropogenic salinization [47], despite the fact that in recent years analyses using isotope tracers have been able to distinguish the sources of river salinization [65]. Although several of these legal cases remain open, in early 2018, the European Commission ruled that the Spanish State illegally provided financial assistance to cover the mine tailing of Vilafruns [66]. In accordance with the ‘Polluter Pays’ principle, these costs should have been paid by the current mining company [66].

### (b) Salinization and human health in the Llobregat catchment

A sizeable share of the controversial public investments destined to ease the impacts of salinization on the Llobregat waters was directed at improving the technology of water purification plants of the Barcelona metropolitan region [63]. Despite the positive impact of the brine sewer in reducing the salinity of the Llobregat waters, and even if the proportion of Cl^−^ to Br^−^ ions in the river was around a satisfactory 500 : 1 [62], the presence of bromide ions had become a serious health issue for Barcelona drinking water [67,68].

During the 1930s, most of the concern about the impact of salinization had focused on taste, along with the impacts of salty water on industry, agriculture and fauna [26]. However, some voices such as the anarchist physician Félix Martí Ibáñez, head of sanitation services in Catalonia during the Spanish Civil War, warned explicitly about the long-term effects that drinking salinized waters could have on human health [69,70]. Martí Ibáñez went into exile after the Francoist victory in the war in 1939, but his concerns were shared by other physicians in the Llobregat region, as revealed by some of the secret reports collected by the dictatorship information services during the 1940s [71].

However, the full extent of this matter only started to be researched during the 1970s, when the first DBPs were identified. The first DBPs studied were THMs, which include chlorinated and brominated compounds [43]. After its discovery, laboratories of water supply systems throughout the world conducted studies to test their presence in their waters. Early studies in both Bremen and Barcelona—whose river waters suffered from the salinization of potash mines—confirmed high concentrations of THMs [67,72]. In both cases, concentrations of bromides originating in the potash mines were identified as precursors contributing to THM formation [67,72].

In water supply systems able to afford the expenditure, costly disinfection methods have been installed to prevent the formation of DBPs or keep them below certain concentrations. In Barcelona, research connecting the presence of THMs in the city's waters to the incidence of bladder cancer contributed to increasing the preoccupation about water quality [73,74]. After several decades of efforts and the approval of strict regulations, membrane technology has been adopted at water purification plants. This technology, more typically applied to desalination, was adopted in order to ensure the proper purification of Llobregat waters and avoid threats to health [37,75]. This improvement, however, has been carried out at the expense of public investments, raising concerns about the transference of private costs to the public sphere. Again, the diverse sources of pollution of the Llobregat, together with the natural salinity of the river waters, contributed to downplaying the responsibility of specific actors for the presence of high concentrations of bromide in the river waters, thus naturalizing the presence of THMs in the region's waters.

## 4. Conclusion

In the Llobregat, natural salinity and the diversity of sources contributing to river salinization has been used since the 1920s to downplay human-induced salinization caused by potash mining. Despite its natural characteristics—a Mediterranean river with a contribution of natural geological salinity in one of its tributaries—the beginning of potash mining in the region during the 1920s soon caused an increase in salt levels and then a socioenvironmental conflict that today has reached the Court of Justice of the EU, where several legal motions remain open. The differences between primary and secondary salinization are key to establishing the liabilities of the mining companies, under the ‘Polluter Pays’ principle, and have been controversial since mines started operating. The social and environmental history of this conflict shows how salinization processes can create long-standing controversies where causal factors supported by scientific evidence are downplayed, similarly to other cases such as climate change where causalities have become a terrain of struggle too difficult for the natural sciences to cover alone. Interdisciplinary analysis, bringing in the social sciences and the humanities, is critical to fully unravel causalities and to design just and sustainable solutions.

Research about the socioenvironmental conflicts caused by salinization and its human health effects has received little attention in comparison to work on the impacts of salinization on fauna and flora. Following a critical social science approach, in this review, we have been interested in exposing how a process of naturalization of human-induced salinization has historically unfolded in the Llobregat river basin. Salinization can be framed not only as a phenomenon affecting river flora and fauna but as a problem of environmental justice as well in which human and non-human actors interacting with each other produce situations of distress, unequal exposure and conflict. The formation of DBPs and the centrality of the latter in the sanitary control of water supply to Barcelona is a good example of the uncertainties faced in the interaction between ions and pollutants. Bromide concentrations, around 500 times smaller than chlorine, are a key precursor of THMs. Paradoxically, its importance to the safety of water supply only emerged once the brine sewer had been built and salinization in the Llobregat waters had decreased significantly. While the option of using desalination technology to guarantee water quality may be possible in a city like Barcelona or other affluent countries, certainly not everybody can afford using desalination technology to purify water, and in many regions of the world disinfected water remains a first priority. Moreover, the example of bromide interactions with organic matter to produce THMs points to the need to expand research on the interaction of salinity and other stressors, in particular, under the postulates of the hydrosocial approach.

The contested character of salinization in the Llobregat catchment also shows the importance of integrating social and environmental history with the natural sciences. The decades of salinization data conserved in archives have a potential use for present day research. By monitoring a significant decrease in water salinization during the Spanish Civil War, the only moment in the last 90 years when potash mines almost stopped operating, the workers of the water company set the grounds to challenge the naturalization arguments of the mining companies (and the Spanish state today). After the war, a new higher legal limit of salinity in the river waters was approved by the Francoist regime, therefore naturalizing the growing human-induced salinity of the river. Nonetheless, data about the salinization of the Llobregat catchment have been collected for decades before isotope tracers permitted distinguishing between natural and human-induced salinization. Archival records can be integrated with current models to develop much-needed research on the different drivers behind river salinization and its importance in the Llobregat waters through time. In exploring the contribution of different drivers to salinity, ions and their mutual interactions and the social and political translation of these processes, social sciences can combine with natural sciences in ways that mutually benefit from an interdisciplinary approach to the analysis and management of environmental problems.

## Data accessibility

This article has no additional data.
